# Clinicopathological analysis of primary refractory diffuse large B‐cell lymphoma treated with rituximab plus cyclophosphamide, doxorubicin, vincristine, and prednisolone chemoimmunotherapy

**DOI:** 10.1002/cam4.4062

**Published:** 2021-06-09

**Authors:** Tomotaka Suzuki, Dai Maruyama, Akiko Miyagi‐Maeshima, Junko Nomoto, Kinuko Tajima, Yuta Ito, Shunsuke Hatta, Sayako Yuda, Shinichi Makita, Suguru Fukuhara, Wataru Munakata, Tatsuya Suzuki, Hirokazu Taniguchi, Koji Izutsu, Yukio Kobayashi, Kensei Tobinai

**Affiliations:** ^1^ Department of Hematology National Cancer Center Hospital Tokyo Japan; ^2^ Department of Pathology National Cancer Center Hospital Tokyo Japan

## Abstract

**Background:**

Approximately 15% of patients with diffuse large B‐cell lymphoma (DLBCL) experience refractory or early relapsed disease after initial rituximab‐containing chemoimmunotherapy is regarded as a primary refractory disease. Although the standard treatment for relapsed DLBCL is high‐dose chemotherapy and autologous stem cell transplantation (HDC‐ASCT), the efficacy of this approach for primary refractory DLBCL is not well understood. We aimed to investigate the clinicopathological characteristics and outcomes of patients with primary refractory DLBCL.

**Methods:**

Sixty‐nine consecutive patients with primary refractory DLBCL who were treated at our institution were categorized as partial responders (partial response to rituximab plus cyclophosphamide, doxorubicin, vincristine, and prednisolone [R‐CHOP] or relapse within 6 months of R‐CHOP) (*n* = 41) or primary progressors (no response to R‐CHOP) (*n* = 28). Survival curves were constructed using the Kaplan–Meier method and compared using the log‐rank test.

**Results:**

At initial diagnosis, 70% of patients had Ann Arbor stage III/IV disease, 56% had non‐germinal center B‐cell‐like type DLBCL, and 42% had double‐expressor lymphoma (MYC and BCL2 expression). The 3‐year overall survival rate was significantly poorer in the primary progressors group than in the partial responders’ group (15% vs. 48%, *p* < 0.001). Four of 17 patients treated with HDC‐ASCT were primary progressors; only one patient survived without relapse. Although double‐expressor lymphoma status did not significantly impact overall survival among all patients (*p* = 0.794), it was identified as an independent poor prognostic factor in HDC‐ASCT‐treated patients (*p* = 0.002).

**Conclusions:**

We identified a subgroup of patients with primary refractory DLBCL who may not benefit from current treatment strategies. Further treatment development is needed to improve the outcomes of these patients.

## Graphical abstract

Sixty‐nine consecutive patients with primary refractory DLBCL who were treated at our institution were clinicopathologically analyzed.We identified a subgroup of patients with primary refractory DLBCL, including primary progressors (no response to R‐CHOP) or those with the double expression of MYC and BCL2, who may not benefit from current treatment strategies.Further treatment development is needed to improve the outcomes of these patients.

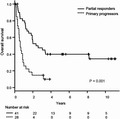

## 1 INTRODUCTION

Rituximab‐containing chemoimmunotherapy (e.g., rituximab plus cyclophosphamide, doxorubicin, vincristine, and prednisolone [R‐CHOP]) is the standard initial treatment for patients with diffuse large B‐cell lymphoma (DLBCL).^1^, ^2^ However, 10.0%–15.0% of patients experience refractory or early relapsed disease, which is regarded as primary refractory DLBCL and generally results in poor outcomes.^3^, ^4^, ^5^

High‐dose chemotherapy and autologous stem cell transplantation (HDC‐ASCT) is the standard of care for patients with relapsed or refractory (rel/ref) DLBCL who respond to salvage therapy.^6^ However, the effectiveness of this strategy in patients with primary refractory DLBCL is uncertain. Furthermore, the clinical outcomes of patients with primary refractory DLBCL who are ineligible for HDC‐ASCT for various reasons, such as older age, poor general condition, or central nervous system (CNS) progression, have not been sufficiently reported.

Several biomarkers, including cell‐of‐origin (COO), the double expression of MYC and BCL2 (double‐expressor lymphoma [DEL]), and *MYC* and *BCL2* rearrangements (double‐hit lymphoma [DHL]), have been identified as adverse prognostic factors for patients with DLBCL.^7^, ^8^, ^9^ However, the prevalence and prognostic significance of these biomarkers in patients with primary refractory DLBCL have yet to be determined.

The purpose of this study was to retrospectively evaluate the clinicopathological characteristics and outcomes of patients with primary refractory DLBCL who were treated at our institution. We aimed to identify patients with primary refractory DLBCL who do not benefit from current treatment strategies and for whom further therapeutic development is needed.

## 2 MATERIALS AND METHODS

### 2.1 Patients

Of the 451 patients with newly diagnosed DLBCL who received R‐CHOP as initial treatment between 2003 and 2015 at the National Cancer Center Hospital (NCCH), 69 consecutive patients who developed primary refractory DLBCL were included in this retrospective analysis. Patients with histology of transformed low‐grade B‐cell lymphoma were excluded. Patients with histology of high‐grade morphology, primary mediastinal large cell lymphoma, or CNS disease at initial diagnosis were also excluded.

Patients with primary refractory DLBCL were categorized as partial responders (partial response [PR] at the end of treatment [EOT] or complete response at the EOT with relapse within 6 months of the last dose of R‐CHOP) (*n* = 41) or primary progressors (disease progression during R‐CHOP or no response at the EOT) (*n* = 28). Patients who received additional, unplanned radiotherapy (RT) for localized residual disease after R‐CHOP were included as partial responders in this study.

The study design was approved by the ethics committee of the National Cancer Center Hospital, prior to commencing this study. Research was conducted in accordance with the international ethical recommendations stated in the Declaration of Helsinki. The need for informed consent was waived due to the retrospective nature of the study.

### 2.2 Salvage therapy

Salvage therapy was administered at the discretion of the attending physician. In general, intensive chemotherapy followed by HDC‐ASCT was planned for patients aged ≤65 years with sufficient organ function. In patients ineligible for HDC‐ASCT, only salvage chemotherapy was performed. Selected patients with localized residual or rel/ref disease received local treatment (mainly RT) as salvage therapy.

### 2.3 Response evaluation

Treatment response was assessed according to the International Workshop Response Criteria (1999).^10^ In patients who underwent fluorine‐18 fluorodeoxyglucose positron emission tomography/computed tomography (PET‐CT), treatment response was assessed according to the 2007 revised response criteria for malignant lymphoma.^11^

### 2.4 Immunohistochemical and fluorescence in situ hybridization analyses

Immunohistochemical and fluorescence in situ hybridization (FISH) analyses were performed using available sections from formalin‐fixed, paraffin‐embedded tissue blocks obtained at initial diagnosis. The COO subtype was determined according to the Hans algorithm.^7^ Immunostaining for MYC (clone Y69; Abcam) and BCL2 (clone 124; Dako) was performed. The cutoff values for positive MYC and BCL2 expression were 40% and 50%, respectively.^9^ FISH analysis for *MYC* rearrangements was performed using an LSI MYC dual‐color break‐apart rearrangement probe (5J9101) (Vysis^®^; Abbott Molecular). FISH analysis for the *IGH*‐*BCL2* rearrangement was performed in *MYC* rearrangement positive patients using an LSI IGH‐BCL2 dual‐color, dual‐fusion translocation probe (5J7101) (Vysis^®^; Abbott Molecular). At least 100 nuclei were counted. Rearrangement was defined as the presence of break‐apart signals of *MYC* and fusion signals of *IGH*‐*BCL2* in ≥10% of nuclei, respectively.

### 2.5 Statistical analysis

PFS and OS were assessed from the time of primary refractory disease. PFS was defined from the starting point until progression or death from any cause, and patients who were still alive and progression‐free at the end of the study period were censored on the date of the last available follow‐up. OS was defined from the starting point until death from any cause, and patients who did not die during the study period were censored on the date of the last follow‐up. Survival curves were constructed using the Kaplan–Meier method, with between‐group comparisons made using the log‐rank test. A *p* < 0.05 was considered statistically significant. All statistical analyses were conducted using EZR (version 1.35; Saitama Medical Center, Jichi Medical University), a graphical user interface for R (The R Foundation for Statistical Computing). Further information on the Materials and Methods can be found in the Supporting Information.

## 3 RESULTS

### 3.1 Patient characteristics

**FIGURE 1:**
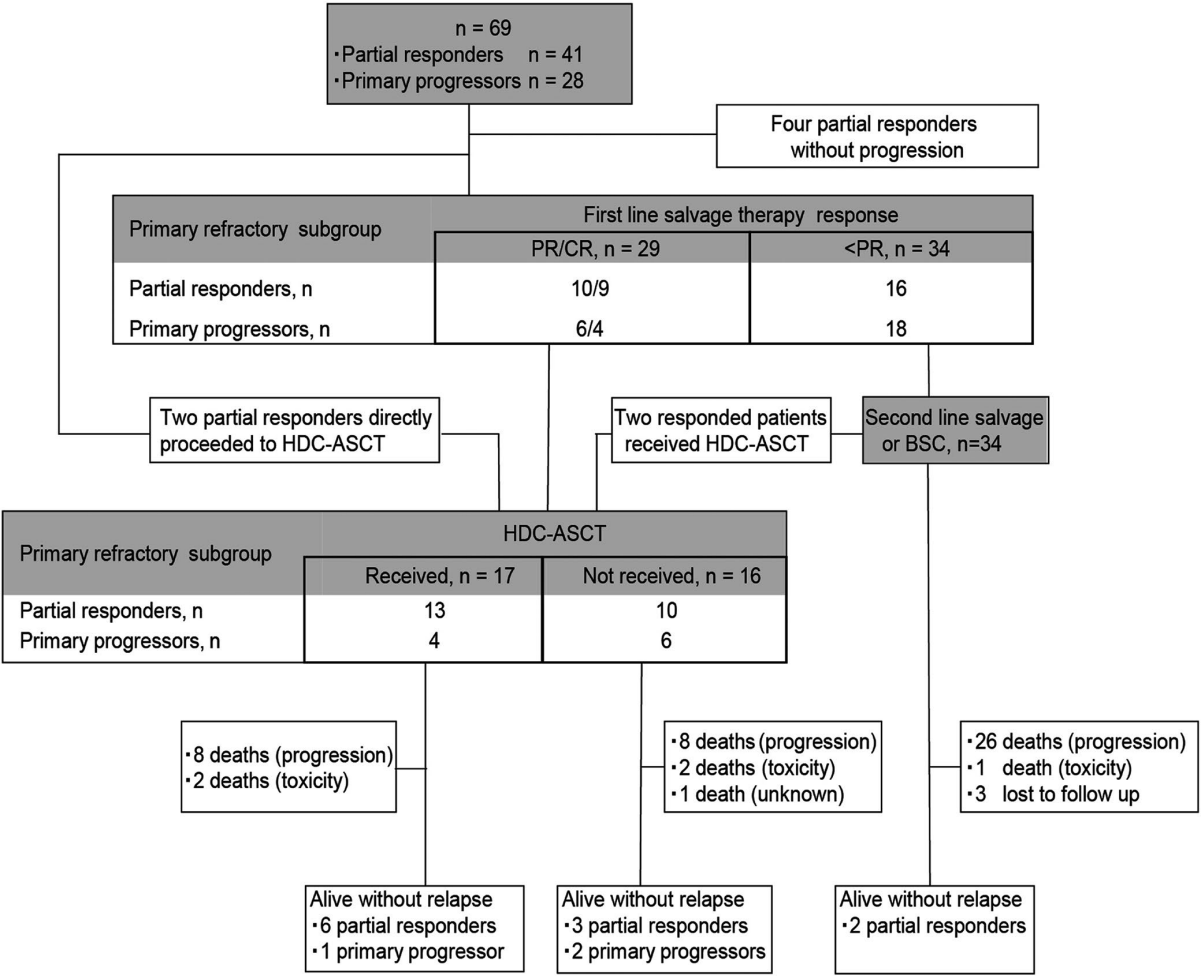
Clinical outcomes of patients with primary refractory diffuse large B‐cell lymphoma (*n* = 69). Abbreviations: ASCT, autologous stem cell transplantation; BSC, best supportive care; CR, complete response; HDT, high‐dose chemotherapy; PR, partial response

Sixty‐nine (15%) of the 451 patients with newly diagnosed DLBCL who received R‐CHOP as initial treatment at the NCCH between 2003 and 2015 were identified as having primary refractory DLBCL. The patient flow chart is shown in Figure 1. The clinicopathological characteristics of the study cohort are summarized in Table 1. The median age at initial diagnosis was 64 (range, 24–82) years. Thirty‐three patients (48%) were male, 55 patients (80%) had elevated lactate dehydrogenase levels, and 48 patients (70%) had Ann Arbor stage III/IV disease. Forty‐three patients (62%) were considered high‐intermediate or high‐risk according to the International Prognostic Index (IPI)^12^ at initial diagnosis. Based on the initial treatment response, 41 patients (59%) were categorized as partial responders (24 patients exhibited PR at the EOT; 17 patients relapsed within 6 months of the last dose of R‐CHOP) and 28 patients (41%) were categorized as primary progressors. Thirty‐nine patients (27 partial responders and 12 primary progressors) were evaluated with PET‐CT at the EOT.

**TABLE 1:** Patient characteristics

Characteristic	All	Partial responders	Primary progressors
	(*n* = 69)	(*n* = 41)	(*n* = 28)
Initial diagnosis			
Age, years, median (range)	64 (24–82)	60 (24–82)	67.5 (33–81)
Sex, male/female, *n*	33/36	17/24	16/12
LDH >UNL, *n* (%)^a^	55 (80)	32 (78)	23 (82)
ECOG PS 2–4, *n* (%)^b^	19 (28)	11 (27)	8 (29)
Ann Arbor stage III/IV, *n* (%)	48 (70)	25 (61)	23 (82)
Extranodal disease (>1), *n* (%)	29 (42)	15 (37)	14 (50)
IPI score, *n* (%)^c^			
0–2	26 (38)	20 (49)	6 (21)
3–5	43 (62)	21 (51)	22 (79)
Immunohistochemistry, *n* (%)			
GCB/non‐GCB^d^	28/35	14/23	14/12
MYC expression	37 (61)	20 (57)	17 (65)
BCL2 expression	44 (73)	27 (79)	17 (65)
MYC and BCL2 expression	25 (42)	15 (44)	10 (40)
Fluorescence in situ hybridization, *n* (%)			
*MYC* rearrangement	8 (19)	1 (3.8)	7 (43)
*MYC* and *BCL2* rearrangements	3 (6.7)	0 (0)	3 (17)
Primary refractory disease			
CNS relapse, *n* (%)^e^	13 (19)	8 (20)	5 (18)
IPI score, *n* (%)			
0–2	43 (62)	30 (73)	13 (46)
3–5	26 (38)	11 (27)	15 (54)

a LDH, lactate dehydrogenase; ULN, upper limit of normal.

b ECOG PS, eastern cooperative oncology group performance status.

c IPI, international prognostic index.

d GCB, germinal center B‐cell‐like.

e CNS, central nervous system.

The COO was determined in 63 patients (91%). Twenty‐eight patients (44%) had germinal center B‐cell (GCB)‐like DLBCL; 35 patients (56%) had non‐GCB DLBCL. The double expression of MYC and BCL2 was evaluated in 59 patients (86%) and confirmed in 25 patients (42%). FISH analysis for *MYC* rearrangements was performed in 56 patients (81%). Eight patients (14%) were positive for *MYC* rearrangements, 34 patients (61%) were negative, and 14 patients (25%) were undetermined. Three out of 45 patients (6.7%) had DHL; all three of these patients were categorized as primary progressors.

The IPI risk at the time of primary refractory DLBCL was low in 24 patients (35%), low‐intermediate in 19 patients (28%), high‐intermediate in 14 patients (20%), and high in 12 patients (18%). CNS disease progression was observed in 13 patients (19%). The disease was localized to the CNS in 11 patients while the other two had concomitant systemic diseases. The median duration from initial diagnosis to CNS progression was 7.9 (range, 1.9–8.8) months. The baseline characteristics of the 13 patients with CNS disease progression are described in Table S1. Although all patients had at least one extranodal disease at initial diagnosis, only one patient had adrenal involvement, which is considered high‐risk for CNS relapse according to the CNS‐IPI.^13^

### 3.2 Salvage therapy and outcomes

Sixty‐three patients (91%) received salvage therapy (systemic chemotherapy with or without RT [*n* = 42] or local treatment only [*n* = 21]). Four patients, who had achieved a PR at the EOT confirmed with PET‐CT, did not show disease progression regardless of further treatment. These four patients showed residual 18F‐fluorodeoxyglucose‐uptake where lymphoma involvement was observed at initial diagnosis; a biopsy of these lesions was not performed. The remaining two patients (partial responders) proceeded directly to HDC‐ASCT without intervening salvage therapy. The salvage therapies are listed in Table 2. The overall response rate (ORR) of the 63 patients who received salvage therapy was 46% (*n* = 29), with 20% (*n* = 13) exhibiting a complete response (CR).

**TABLE 2:** Salvage therapy according to age

Salvage therapy	Age ≤65 years	Age >65 years
	(*n* = 34)	(*n* = 29)
Systemic chemotherapy, *n* ^a^	24	18
ESHAP^b^	9	1
CODOX‐M/IVAC^c^	6	0
IVAC	2	0
DHAP^d^	2	0
EPOCH^e^	1	3
ICE^f^	1	1
CMOPP^g^	0	8
Other	3	5 (4 trials)
Local treatment, *n*	10	11
Radiation therapy for CNS disease^h^	4	6
Radiation therapy for extra‐CNS disease	5	5
Total gastrectomy	1	0

a Systemic chemotherapy performed with or without monoclonal anti‐CD20 antibody.

b ESHAP, etoposide, methylprednisolone, high‐dose cytarabine, and cisplatin.

c CODOX‐M, cyclophosphamide, doxorubicin, vincristine, and high‐dose methotrexate; IVAC, ifosfamide, etoposide, and high‐dose cytarabine.

d DHAP, dexamethasone, high‐dose cytarabine, and cisplatin.

e EPOCH, etoposide, prednisolone, vincristine, cyclophosphamide, and doxorubicin.

f ICE, ifosfamide, carboplatin, and etoposide.

g CMOPP, cyclophosphamide, vincristine, prednisolone, and procarbazine.

h CNS, central nervous system.

Thirty‐four of the 37 patients aged ≤65 years received salvage therapy (partial responders [*n* = 21] and primary progressors [*n* = 13]). Of these, 20 (59%) (partial responders [*n* = 14] and primary progressors [*n* = 6]) achieved an objective response. Thirteen patients with an objective response were treated with HDC‐ASCT. The remaining patients were not treated with HDC‐ASCT because of successful local treatment (*n* = 5) or poor general condition (*n* = 2). Combining patients with an objective response (*n* = 13) with patients who responded to second‐line salvage therapy (*n* = 2) and partial responders who received upfront HDC‐ASCT (*n* = 2), a total of 17 patients (partial responders [*n* = 13] and primary progressors [*n* = 4]) were treated with HDC‐ASCT.

The conditioning regimens consisted of ranimustine, cyclophosphamide, etoposide, and carboplatin (MCEC)^14^ in 14 patients and melphalan, cyclophosphamide, etoposide, and dexamethasone (LEED)^15^ in three patients. Fifteen patients (PR [*n* = 10] and CR [*n* = 5] before HDC‐ASCT) achieved CR; six patients relapsed within 6 months of HDC‐ASCT. In total, seven patients, including only one primary progressor, survived for >3 years.

Allogeneic hematopoietic stem cell transplantation (HSCT) with a reduced‐intensity conditioning regimen was performed in five patients, three of whom had received prior HDC‐ASCT. Two patients died of early transplant‐related adverse events. Responses were assessed in the remaining three patients. One patient achieved CR and the remaining two patients had progressive disease. However, since the patient who achieved CR died of late transplant‐related adverse events, none of the patients who received allogeneic HSCT were still alive. The ORR among patients aged >65 years who received salvage chemotherapy (*n* = 18), including two patients who received allogeneic HSCT, was 11%, with only one patient surviving for >3 years.

In the 13 patients with CNS progression, salvage therapy was performed as follows: whole‐brain RT with or without intrathecal chemotherapy in 10 patients, whole‐brain RT followed by systemic chemotherapy and HDC‐ASCT in one patient, whole‐brain RT followed by chemotherapy for extra‐CNS disease in one patient, and chemotherapy followed by HDC‐ASCT in one patient. Nine patients (69%) achieved an objective response. Four patients had relapsed or progressive disease (CNS disease [*n* = 2] and extra‐CNS disease [*n* = 2]), and three patients died of treatment‐related adverse events. Only two patients (15%), one who received chemotherapy followed by HDC‐ASCT and one who received whole‐brain RT, were alive at last follow‐up (40 and 38 months, respectively).

Twenty‐one patients (partial responders [*n* = 13] and primary progressors [*n* = 8]), including 10 patients with CNS progression, received local treatment only as salvage therapy. The ORR was 62% (*n* = 13); 43% for patients with CR (*n* = 9). In primary progressors, there was evidence of progression inside (*n* = 4) and outside (*n* = 1) the irradiated fields. In partial responders, there was also evidence of progression inside (*n* = 2) and outside (*n* = 7) the irradiated field. Five patients (partial responders [*n* = 3] and primary progressors [*n* = 2]) survived for >3 years.

### 3.3 Survival analysis

**FIGURE 2:**
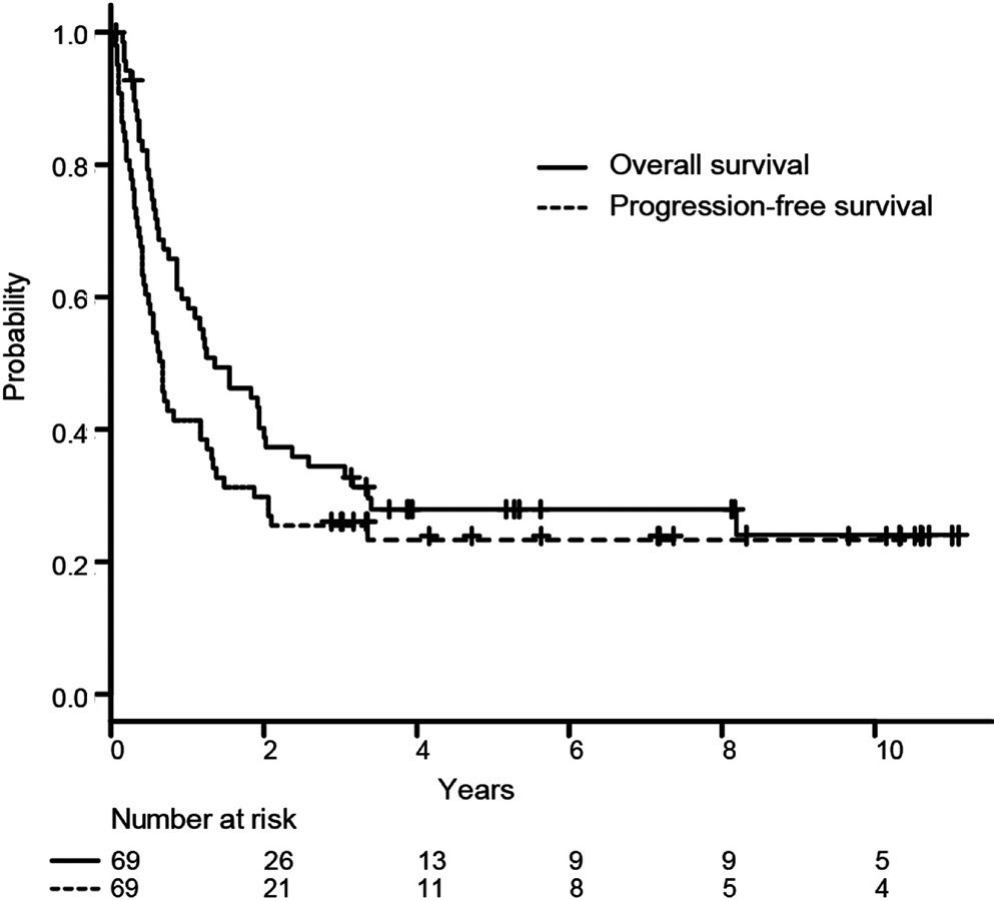
Kaplan–Meier curves of overall survival (solid line) and progression‐free survival (dashed line) in patients with primary refractory diffuse large B‐cell lymphoma (*n* = 69)

**FIGURE 3:**
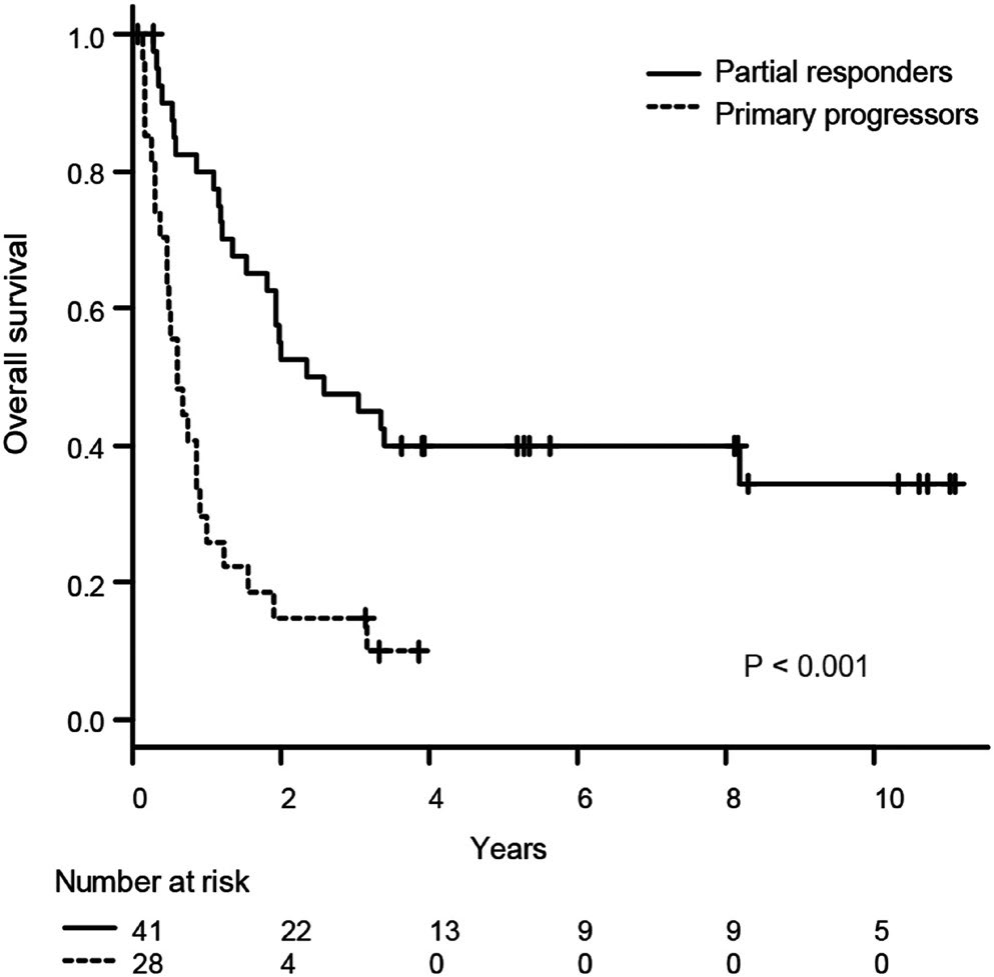
Kaplan–Meier curves of overall survival stratified by response to initial treatment. Partial responders are represented by the solid line; primary progressors are represented by the dashed line

The median follow‐up time of survivors since the time of primary refractory DLBCL was 65 (range, 0.8–135) months. In all 69 patients, the 3‐year PFS and OS rates were 26% (95% confidence interval [CI]: 16%–37%) and 34% (95% CI: 23%–46%), respectively (Figure 2). The 3‐year PFS and OS rates of the 17 patients treated with HDC‐ASCT were 41% and 47%, respectively. Patients in the primary progressors group had a significantly poorer prognosis than those in the partial responders’ group (3‐year OS: 15% vs. 48%, respectively; *p* < 0.001; Figure 3). A higher IPI risk at the time of primary refractory DLBCL was also associated with a poorer prognosis (Figure S1). CNS progression was not a significant prognostic factor (data not shown).

**FIGURE 4:**
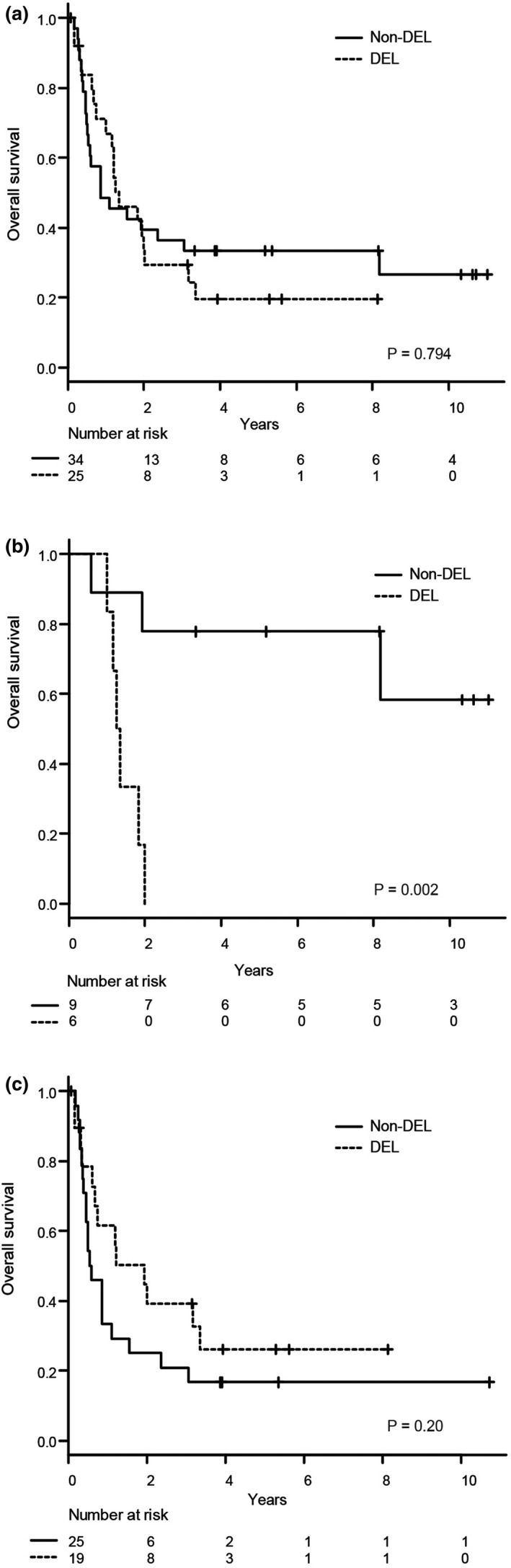
Kaplan–Meier curves of (A) overall survival according to the status of the double expression of MYC and BCL2 (double‐expressor lymphoma [DEL]) in patients with known DEL status (*n* = 59) and (B) and (C) overall survival in patients treated with and without high‐dose chemotherapy and autologous stem cell transplantation stratified according to DEL status

In the 59 patients evaluated for the double expression of MYC and BCL2, DEL status did not significantly impact OS (Figure 4A). DEL status was identified as an adverse factor for OS in patients treated with HDC‐ASCT (*n* = 15), including three patients who subsequently received allogeneic HSCT (*p* = 0.0023; Figure 4B), but not in those who were not treated with HDC‐ASCT (*n* = 44), including two patients who received allogeneic HSCT (Figure 4C). *MYC* rearrangements and COO subtype (GCB‐like vs. non‐GCB) were not significant prognostic factors (data not shown).

## 4 DISCUSSION

Herein, we evaluated the clinicopathological characteristics and outcomes of 69 patients with primary refractory DLBCL. In the current study, 15% of patients with newly diagnosed DLBCL had primary refractory disease to R‐CHOP. These patients had a poor prognosis (3‐year PFS and OS rates: 26% and 34%, respectively). Primary progressors had extremely poor outcomes, regardless of HDC‐ASCT. Among the 13 primary progressors aged ≤65 years, four were treated with HDC‐ASCT, resulting in long‐term survival in only one patient. The proportions of patients with DEL and DHL were unexpectedly low in this group (42% and 6.7%, respectively). Although DEL status was not a prognostic factor among all patients, it may be a prognostic factor for OS when restricted to patients treated with HDC‐ASCT.

The standard of care for relapsed DLBCL is salvage therapy followed by HDC‐ASCT. This was first shown by the PARMA study.^6^ However, the effectiveness of this strategy for primary refractory DLBCL has not been sufficiently evaluated, because only patients with relapsed disease after achieving CR were included in the PARMA study.^6^ In the CORAL study,^16^ patients with rel/ref DLBCL were randomly assigned to one of either two salvage chemotherapies including rituximab, and responding patients received HDC‐ASCT. In the ORCHARRD study,^17^ patients with rel/ref DLBCL were randomly assigned to salvage chemotherapy comprising rituximab or ofatumumab. The CORAL and ORCHARRD studies^16^, ^17^ both demonstrated that patients with rel/ref DLBCL to first‐line rituximab‐containing chemoimmunotherapy (within 12 months) had a poor prognosis (3‐year PFS rates: 23% and <20%, respectively). Furthermore, in a single‐center retrospective study conducted at the Memorial Sloan‐Kettering Cancer Center,^18^ 82 DLBCL patients with less than CR to first‐line rituximab‐containing chemoimmunotherapy underwent salvage therapy with the intent to proceed to HDC‐ASCT. The 3‐year PFS rates differed considerably between patients who achieved PR and those who achieved less than PR (same definition as primary progressors in our study) (49% and 17%, respectively). The results of our study and the Memorial Sloan‐Kettering Cancer Center^18^ suggest that primary progressors may receive little benefit from current treatment strategies.

To the best of our knowledge, this is the first report to evaluate the prognostic impact of DEL status solely focused on primary refractory DLBCL. Although DEL status did not significantly impact OS among all patients, it was a poor prognostic factor in patients treated with HDC‐ASCT. Recently, similar reports have been made by the Canadian Cancer Trials Group. The NCIC‐CTG LY. 12 trial^19^ compared the efficacy of two salvage chemotherapy regimens followed by HDC‐ASCT in patients with rel/ref aggressive non‐Hodgkin lymphoma. In a subgroup analysis of the NCIC‐CTG LY. 12 trial,^20^ the 3‐year OS rate post‐HDC‐ASCT was significantly lower in patients with DEL than in those without DEL (55% vs. 88%, respectively). In addition, Herrera et al.^21^ reported that DEL status was a poor prognostic factor for PFS in rel/ref DLBCL patients treated with HDC‐ASCT. The above two studies and our own might suggest that the effectiveness of HDC‐ASCT is limited in patients with DEL, although this remains inconclusive due to the retrospective nature and/or subgroup analyses.

Several novel immunotherapies, including autologous anti‐CD19 chimeric antigen receptor (CAR) T‐cell therapy, anti‐CD20 × anti‐CD3 bispecific antibody, and CD79b‐targeted antibody‐drug conjugate, have been studied for the treatment of rel/ref B‐cell lymphoma.^22^, ^23^, ^24^, ^25^ In pivotal phase II studies, anti‐CD19 CAR T‐cell therapy demonstrated promising efficacy for rel/ref DLBCL after initial treatment^23^ or the last line of treatment^22^ (ORR: 86% and 40%, respectively). Phase III studies comparing anti‐CD19 CAR T‐cell therapy and the current standard of care with salvage therapy followed by HDC‐ASCT as second‐line therapy for rel/ref DLBCL (NCT03570892, NCT03575351, and NCT03391466) are currently being conducted. These studies are expected to reveal the role of anti‐CD19 CAR T‐cell therapy in patients with refractory disease, whose benefit from salvage therapy followed by HDC‐ASCT may be limited.

In the current study, 13 patients who developed CNS progression (median duration, 7.9 months since initial diagnosis) had primary refractory DLBCL. In line with our study, it has been reported that the median time to CNS progression was 6.7–7.2 months since initial diagnosis in newly diagnosed DLBCL patients.^13^ Therefore, the establishment of preventive measures for CNS progression is an important factor in overcoming primary refractory DLBCL.

There are several limitations to the current study. First, this was a retrospective study with a relatively small sample size. Second, immunohistochemical analysis could not be performed in all patients, which may have resulted in selection bias. Finally, response was not evaluated with the Lugano 2014 response criteria.^26^ However, despite these limitations, we believe that our study, which investigated the clinicopathological features of DLBCL patients primary refractory to R‐CHOP, is of value because R‐CHOP remains the standard of care for untreated DLBCL and, to date, no other treatment strategies have shown superiority to R‐CHOP in randomized phase III studies.^27^, ^28^, ^29^, ^30^, ^31^, ^32^, ^33^

In conclusion, our study presents a comprehensive overview of primary refractory DLBCL, which is clinicopathologically heterogeneous. RT for localized disease and HDC‐ASCT remain potentially curative approaches for selected patients. However, certain patient groups, including primary progressors, those with DEL, and those with CNS progression, had unsatisfactory clinical outcomes following conventional treatment strategies. Treatment strategies with novel agents, such as novel immunotherapies or molecular targeted drugs, may be needed to improve the outcomes of these patients.

## CONFLICT OF INTEREST

Dr. Maruyama reports grants and personal fees from Ono Pharmaceutical, Celgene, Takeda Pharmaceutical, Janssen Pharmaceutical, Bristol‐Myers Squibb and Chugai Pharmaceutical, personal fees from Eisai, Kyowa Hakko Kirin, Zenyaku Kogyo, SYNMOSA BIOPHARMA and NIPPON SHINYAKU, grants from Merck, Amgen, Astellas BioPharma, Astellas Pharma, Sanofi, Novartis Pharma, Otsuka Pharmaceutical, outside the submitted work. Dr. Makita reports personal fees from Celgene/BMS, Chugai Pharmaceutical, Daiichi‐Sankyo, Eisai, Symbio, and Takeda, outside the submitted work. Dr. Izutsu reports grants and personal fees from Kyowa Hakko Kirin, grants from Chugai Pharmaceutical, and Zenyaku Kogyo, outside the submitted work. Dr. Tobinai reports personal fees from Eisai, Takeda, Mundipharma, HUYA Bioscience International, Kyowa Hakko Kirin, Celgene, Chugai Pharma, Ono Pharmaceutical, Yakult, Daiichi Sankyo, and Zenyaku Kogyo outside the submitted work.

## Supporting information

Fig S1

Table S1

Supplementary Material

## Data Availability

Inquiries for data should be directed to dmaruyam@ncc.go.jp. The date will be available for achieving aims in the approved proposal.
