# Supplementary material for: Clinicopathological analysis of primary refractory diffuse large B‐cell lymphoma treated with rituximab plus cyclophosphamide, doxorubicin, vincristine, and prednisolone chemoimmunotherapy
Source: Cancer Med. 2021 Jun 9;10(15):5101–9. doi: 10.1002/cam4.4062 (PMC8335825; doi:10.1002/cam4.4062)
Supplement: Supplementary file 1 — Fig S1 [file CAM4-10-5101-s003.docx]

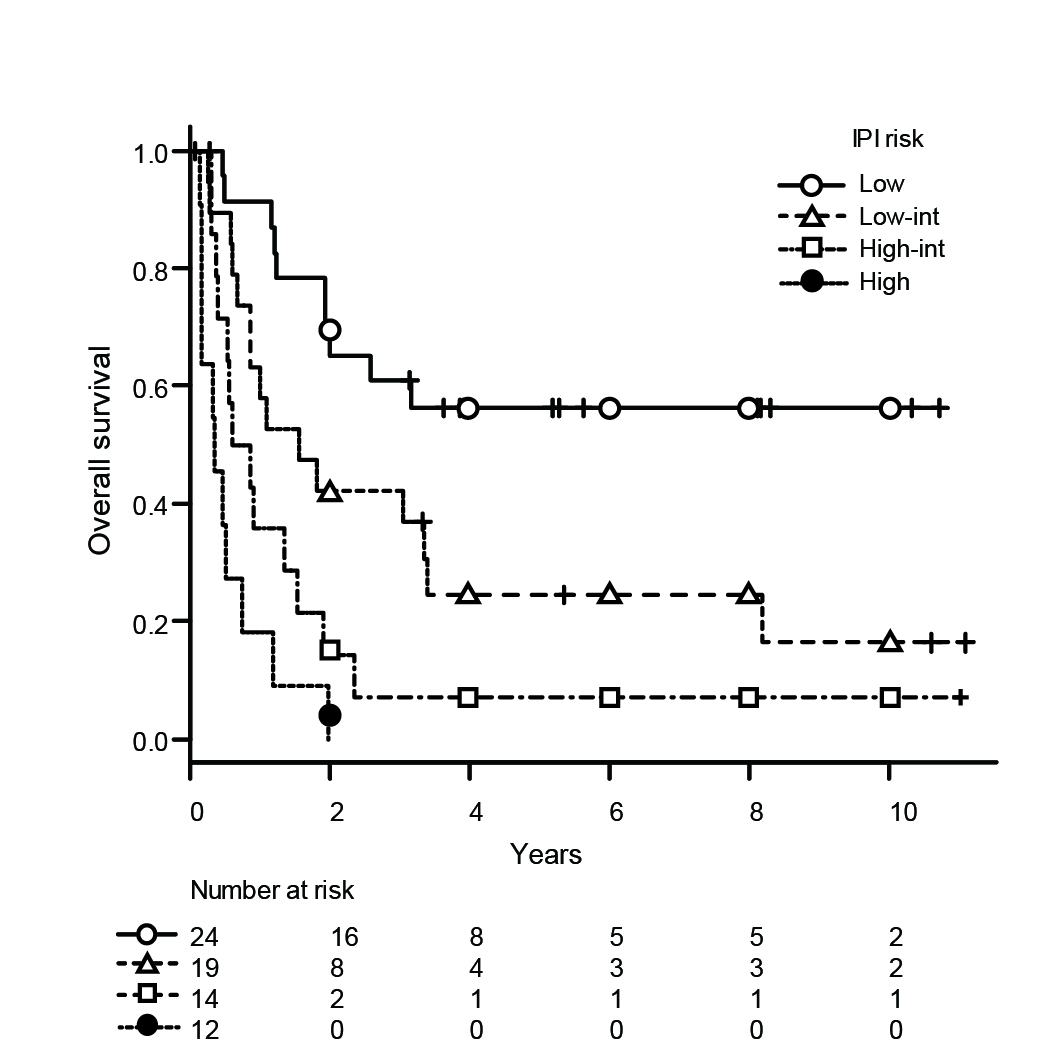


**Figure S1.** Kaplan-Meier curves of overall survival according to the International Prognostic Index risk group at the time of primary refractory DLBCL. The low-, low-intermediate-, high-intermediate-, and high-risk groups are represented by the open circles (solid line), triangles (dashed line), squares (dashed line), and solid circles (dashed line), respectively. Abbreviations: IPI, International Prognostic Index; int, intermediate
