# Supplementary material for: Clinicopathological analysis of primary refractory diffuse large B‐cell lymphoma treated with rituximab plus cyclophosphamide, doxorubicin, vincristine, and prednisolone chemoimmunotherapy
Source: Cancer Med. 2021 Jun 9;10(15):5101–9. doi: 10.1002/cam4.4062 (PMC8335825; doi:10.1002/cam4.4062)
Supplement: Supplementary file 2 — Table S1 [file CAM4-10-5101-s001.docx]

**Table S1.** Baseline characteristics of patients with CNS progression^†^

| Baseline characteristic | Patients with CNS^‡^ progression  (n = 13) |  |
| --- | --- | --- |
| Age, years, median (range) | 68 (24–80) |  |
| Sex, male/female, n | 8/5 |  |
| LDH >UNL^§^, n (%) | 12 (92) |  |
| ECOG PS^¶^ 2–4, n (%) | 5 (39) |  |
| Ann Arbor stage III/IV, n (%) | 8 (62) |  |
| Extranodal disease (≥2), n (%) | 6 (46) |  |
| Site of extranodal disease |  | |
| Bone marrow or bone, n | 5 | |
| Digestive tract, n | 4 | |
| Mammary gland, n | 2 | |
| Peritoneum, n | 2 | |
| Adrenal gland, n | 1 | |
| Other sites^††^, n | 6 | |
| CNS-IPI^‡‡^score, n (%) |  | |
| 0–1 | 2 (15) |  |
| 2–3 | 6 (46) |  |
| 4–6 | 5 (39) |  |
| Immunohistochemistry, n (%) |  |  |
| CD5 expression | 1 (7.7) |  |
| GCB/non-GCB^§§^ | 4/9 |  |
| MYC expression (n = 12) | 6 (50) |  |
| BCL2 expression (n = 12) | 8 (67) |  |
| MYC and BCL2 expression (n = 11) | 3 (27) |  |

^†^None of the patients received prophylaxis (intrathecal chemotherapy/high-dose methotrexate) for CNS relapse

^‡^CNS, central nervous system

^§^LDH, lactate dehydrogenase; ULN, upper limit of normal

^¶^ECOG PS, Eastern Cooperative Oncology Group performance status

^††^Other sites were as follows, nasal and oral cavity (n=1), muscle (n=1), skin (n=1), lung (n=1) pleura (n=1) and uterus (n=1)

^‡‡^CNS-IPI, central nervous system International Prognostic Index

^§§^GCB, germinal center B-cell-like
