# Supplementary material for: Clinicopathological analysis of primary refractory diffuse large B‐cell lymphoma treated with rituximab plus cyclophosphamide, doxorubicin, vincristine, and prednisolone chemoimmunotherapy
Source: Cancer Med. 2021 Jun 9;10(15):5101–9. doi: 10.1002/cam4.4062 (PMC8335825; doi:10.1002/cam4.4062)
Supplement: Supplementary file 3 — Supplementary Material [file CAM4-10-5101-s002.docx]

**Materials and Methods Supporting Information**

***Patients***

Patients with transformed low-grade B-cell lymphoma were excluded. This is because this retrospective study aimed to evaluate the clinicopathological characteristics of patients with *de novo* diffuse large B-cell lymphoma (DLBCL) treated with standard rituximab plus cyclophosphamide, doxorubicin, vincristine, and prednisolone (R-CHOP) chemotherapy, the treatment goal of which is cure. In addition, patients with transformed B-cell lymphoma could develop refractory or relapsed disease, which met the criteria of primary refractory disease but with a low-grade B-cell lymphoma component only; this is not considered clinically equivalent to the primary refractory disease with an aggressive-lymphoma component.

The histology of high-grade morphology was B-cell lymphoma, unclassifiable, and with features intermediate between DLBCL and Burkitt lymphoma according to the 2008 World Health Organization criteria.

***Primary refractory disease definition***

Patients with primary refractory DLBCL were defined as either partial responders (partial response at the end of treatment [EOT] or complete response at the EOT with relapse within 6 months of the last dose of R-CHOP) or primary progressors (disease progression during R-CHOP or no response at the EOT).

The definition of primary refractory disease has not been clearly established. The date of initial diagnosis was not used as the starting point for assessing the time to recurrence because the duration from initial diagnosis to treatment largely differs among patients. In order to include patients with purely refractory disease to R-CHOP, a relapsed disease within 6 months of the last dose of R-CHOP was defined as primary refractory.

***Fluorescence in situ hybridization analyses***

*BCL6 rearrangement* was not evaluated because its significance was considered to be limited in this study; only three patients had both *MYC* and *BCL2* rearrangements.

***Statistical analysis***

The time of primary refractory disease was defined as either of the day when i) response at the EOT was confirmed as partial response (PR) or less than PR, or ii) progression or relapse was confirmed objectively.
